# Evaluation of Bed Depth Reduction, Media Change, and Partial Saturation as Combined Strategies to Modify in Vertical Treatment Wetlands

**DOI:** 10.3390/ijerph18094842

**Published:** 2021-05-01

**Authors:** Ismael Vera-Puerto, Hugo Valdés, Christian Correa, Valeria Perez, Roberto Gomez, Erica Alarcon, Carlos Arias

**Affiliations:** 1 Centro de Innovación en Ingeniería Aplicada, Departamento de Obras Civiles, Facultad de Ciencias de la Ingeniería, Universidad Católica del Maule, Av. San Miguel 3605, Talca 3480112, Chile; ivera@ucm.cl (I.V.-P.); clcorrea@ucm.cl (C.C.); ealarconb@ucm.cl (E.A.); 2 Centro de Innovación en Ingeniería Aplicada, Departamento de Computación e Industrias, Facultad de Ciencias de la Ingeniería, Universidad Católica del Maule, Av. San Miguel 3605, Talca 3480112, Chile; hvaldes@ucm.cl; 3 Escuela de Ingeniería en Construcción, Departamento de Obras Civiles, Facultad de Ciencias de la Ingeniería, Universidad Católica del Maule, Av. San Miguel 3605, Talca 3480112, Chile; valerya_xyu@hotmail.com (V.P.); ro.ber.to.style@hotmail.com (R.G.); 4 Department of Biology-Aquatic Biology, Aarhus University, Ole Worms Allé 1, 8000 Aarhus C, Denmark; 5 WATEC Aarhus University Centre for Water Technology, NyMunkegade, Bldg. 1521, 8000 Aarhus C, Denmark

**Keywords:** nutrients, partial saturation, vertical subsurface flow treatment wetlands, zeolite

## Abstract

The aim of this work was to evaluate the performance of vertical subsurface flow treatment wetlands (VSSF TWs) for treating rural domestic wastewater when strategies such as bed depth reduction and media change are used in combination with bottom saturation. Two treatment wetland systems were implemented: normal (VF-N), with a bed depth of 1.0 m, and modified (VF-M), with a bed depth of 0.5 m and a bottom layer of natural zeolite. *Schoenoplectus californicus* was used as experimental plant. These two treatment systems were operated at a hydraulic loading rate of 120 mm/d in two phases. Phase I did not use bottom saturation, while Phase II involved a bottom saturation of the zeolite layer of the VF-M system. The results show that bed depth reduction did not have a significant effect (*p* > 0.05) in terms of organic matter, solids, and ammonium removal. Conversely, it had a significant influence (*p* < 0.05) on phosphate as well as a negative effect on pathogen removal. This influence could be explained by initial media capacity for phosphorus removal and filtration importance in the case of pathogens. Partial saturation only had a positive influence on total nitrogen removal. The addition of a bottom layer of natural zeolite showed no positive effect on nutrient removal. The plant showed adaptation and positive development in both VF-N and VF-M. The water balance showed that water loss was not influenced by bed depth reduction. Therefore, according to the previous results, a combination of the proposal modifications to VSSF TWs can be introduced for treating rural domestic wastewater.

## 1. Introduction

Vertical subsurface flow treatment wetlands (VSSF TWs) with intermittent application, which are the most commonly models employed, have been shown to be effective for total suspended solids (TSS) and organic matter (biological oxygen demand at 5 days (BOD_5_) and chemical oxygen demand (COD)) removal, exhibiting efficiencies of between 35 and 95 % when they are applied in domestic or municipal wastewater treatment [1]. However, removal efficiency is lower (usually below 60%) in the case of total nitrogen (TN) and total phosphorus (TP) [1,2,3].

Nitrogen removal by VSSF TWs depends on the efficiency of biological processes such as ammonification (organic N transformed to ammonium (NH_4_^+^-N)), nitrification (NH_4_^+^-N to nitrate (NO_3_^−^-N)) and denitrification (NO_3_^−^-N to N_2_ gas) [4]. These processes are influenced by design and operational conditions, such as loading rates (hydraulic and nitrogen), temperature, media size and nature in beds, plants and operative schemes. [5,6,7]. Between 70 and 90% of the TN in municipal wastewater is ammonium [8], and so the initial step of nitrification is key for TN removal at the end of the process. Therefore, oxidative conditions, above +100 mV, that are normally present in VSSF TWs, ensuring aerobic conditions, besides the presence of nitrifying bacteria, and sufficient alkalinity, favor the nitrification process, with ammonium removal efficiencies above 50% [9]. However, the denitrification process is not completely developed in unsaturated pulse fed VSSF, affecting TN removal [10]. The denitrification process is disrupted by the lack of carbon sources and anoxic conditions [11]. Solutions to improve TN removal in VSSF TWs follow two strategies: feeding carbon by external sources such as lignocellulosic waste [12] or modifications in operation such as different feeding strategies and bottom saturation [10,13]. In terms of previously commented strategies, bottom saturation perhaps be the mostly likely to be implemented on full scale VSSF TWs.

Phosphorus removal by VSSF TWs can be achieved by two pathways, a biological pathway by assimilation/accumulation into plants (and then, they have to be harvested) and a physical–chemical pathway by precipitation and adsorption onto the filling media [14]. Considering that phosphate (PO_4_^−3^-P) represents more than 60% of the total phosphorus (TP) present in domestic or municipal wastewater [14], its removal is the most effective way to remove TP. In terms of support media, gravel and sand have traditionally been used as support in subsurface wetlands [15]. Gravel and sand have low P adsorption capacities that vary between 0.03 and 0.05 gP/kg [16]. For this reason, adsorbent materials such as alunite, bauxite, blast furnace slag, calcite, dolomite, Filtralite^®^, fly ash, HeloFIR^®^, LECA, limestone, maerl, marble, norlite, ochre stone, opoka, peat, Polonite^®^, sea shells, vermiculite, zeolite and wollestonite have all been proposed as support media for plants in subsurface wetlands [17] but have shown limited success.

Natural zeolites stand out among the different adsorbent media for their abundance, low price, and regenerative capabilities [18]. Natural zeolites are porous minerals with important physicochemical properties such as cation exchange, molecular sieve, catalysis and sorption [19]. Natural zeolite has the capacity to adsorb phosphate through cation exchange and precipitation as well as, increase the removal of ammonium by the ion exchange created by the negative charge on their surface. This material has an adsorption capacity of 0.3 to 2.5 gP/kg, which is 10 to 100 times higher than gravel and sand [16,20]. Recently, Vera-Puerto et al. [21] described Chilean natural zeolite of a size of 1.5–3.0 mm and a maximum adsorption capacity of up to 0.08 mg/g for PO_4_^−3^-P and 1.58 mg/g for NH_4_^+^-N. In addition, Vera et al. [14] determined that the use of Chilean natural zeolites as a support media in a TW significantly increases (*p* < 0.05) PO_4_^−3^-P removal under aerobic conditions (ORP > +100 mV) at a rate of between 20 and 50%. Araya et al. [4] also determined that Chilean natural zeolites used as TW support media under aerobic conditions (ORP > +100 mV) significantly increase (*p* < 0.05) NH_4_^+^ removal efficiency by up to 70%.

When applying natural zeolite to VSSF TWs the contact time between the wastewater and the zeolite must be increased in order to augment its adsorption capacity of phosphate and ammonium and therefore TN and TP removal. In this way, Silveira et al. [10] proposed bottom saturation (partial saturation of VSSF TWs) as a way to increase the contact time and improve denitrification in VSSF TWs. Their results showed that the variation in saturation height from 0.15 m to 0.25 m significantly modified (*p* < 0.05) the effluent concentration of NO_3_^−^. However, they did not use adsorbent materials such as natural zeolites in the bottom saturated part. This combined strategy (bottom saturation + adsorbent materials) could be interesting considering the potential of local resources, such as Chilean natural zeolites, to increase phosphate and ammonium removal and, therefore, TN and TP removal.

VSSF TWs contribute to disinfection, with removals of between one and three log-units of fecal coliforms or *Escherichia coli* (*E. coli*) [11,22]. In TWs filtration, entrapment, and sedimentation are cited as important processes in the removal of relatively large amounts of pathogens [23]. Furthermore, Headly et al. [22] indicate that the combination of sand media, loading, and vegetation requires less depth to achieve a given effluent *E. coli* concentration. However, the same authors indicate that further research is needed to validate this claim.

Traditionally, VSSF TWs have effective bed depths of between 0.35 m and 1.5 m, although it is common to use depths close to 1.0 m [9]. However, the highest bacterial activity for processing the different pollutants in wastewater occurs in the first 0.2 m (measured from top to bottom) of the wetland [1]. Therefore, reduced depths ranging from 0.08 to 0.6 m have been proposed [24]. This reduction in bed depth would reduce construction costs. However, the combination of reduction in bed depth and strategies such as bottom saturation with adsorbent material require further study in order to optimize the use of VSSF TWs for domestic or municipal wastewater treatment.

This paper aims to consider this information in order to evaluate the performance of vertical subsurface VSSF TWs, when strategies such as bed depth reduction and media change are used in combination with a bottom saturation, for the treatment of rural domestic wastewater.

## 2. Materials and Methods

### 2.1. Influent

Domestic wastewater was used as the influent for feeding the experimental system. Table 1 shows its characteristics. The influent was obtained from effluents to septic tank from a single household of six inhabitants. The influent was transported to a pumping well and subsequently pumped to the experimental system.

The concentration in Table 1 indicates that the influent can be considered “moderated sewage” [8]. However, the NH_4_^+^-N average concentration exceeds 40%, the maximum concentration of 30 mg/L for this classification. This can be explained by process of ammonification developed within the septic tank. Additionally, the TSS influent concentration had a standard deviation above the average concentration. This can be explained by the fact that the sewage was manually extracted which could have resulted in sediment being extracted from the fund of the pit.

### 2.2. Experimental Setup

**Figure 1:**
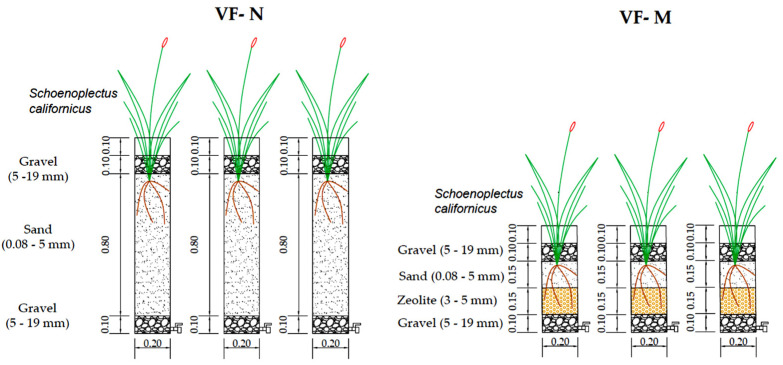
Characteristics of experimental mesocosm VSSF TWs. Dimensions are in meters with exception of numbers inside parentheses that specify grain size in mm.

Six experimental units of VSSF TWs mesocosm were constructed and operated. They were isolated from rain in a room without temperature control and covered by a translucent roof to allow natural light; this location was near the city of Talca (Maule Region, Central Valley, Chile). The VSSF TWs were built using 0.2 m diameter PVC pipes and planted with *Schoenoplectus californicus* [14,25]. The experimental setup was divided into two treatment lines: three VSSF TWs with a total bed depth of 1.0 m (VF-N) and three VSSF TWs with a total bed depth of 0.5 m (VF-M). Bed depth was reduced in VF-M, taking into account that the highest bacterial activity for processing the different pollutants in wastewater occurs in the first 0.2 m [1]. The VSSF TWs were filled with 0.1 m gravel layer (Ø, 5–19 mm) at both the top and bottom. For the VF-N, a layer of sand (Ø, 0.08–5.0 mm) was used as the support media with a depth of 0.8 m. For VF-M, the support media was divided into two layers, a 0.15 m sand (Ø, 5–19 mm) layer followed by a 0.15 m zeolite (Ø, 3–5 mm) layer. The sand used in all VSSF TWs had a d_60_ of 1.2 mm, a d_10_ of 0.25 mm, and the uniformity coefficient (U = d_60_/d_10_) of 4.8. This sand was in agreement with the proposed values of sand rate percolation (SRP), between 45–75 s [15]. Zeolite was supplied by Zeolitas del Maule, a local supplier. Zeolite was employed in VF-M to evaluate its performance for water quality in effluents taking into account its adsorption capacity. Figure 1 presents the characteristics of the mesocosm VSSF TWs.

### 2.3. Operational and Monitoring Strategy

A theoretical hydraulic loading rate (HLR) of 120 mm/d was applied to the VSSF TWs. The HLR was defined after considering a contribution of 100 L/inhab*d [26] applied to an area of approximately 0.85 m^2^ for instantaneous HLR (recommended for design, 3.0 m^2^/inhab for the total VSSF-TWs system). The HLR was applied in twelve pulses per day according to recommendations by Olsson [27], Stefanakis et al. [1] and Brix and Arias [9]. A model FPP-V3 peristaltic pump (Biobase, Jinan, China) was used to feed the two treatment lines. A five-day loading period and ten-day resting period was employed as operational strategy in each treatment line according to recommendations by Stefanakis et al. [1]. The operation was divided into two phases: Phase I, no bottom saturation, over three months for the two systems and, Phase II, bottom saturation only in the zeolite layer for VF-M, over three months. Zeolite layer was saturated to evaluate if adsorption capacity of the material can be enhanced by increasing contact time with the wastewater, or if the bottom saturation modifies the oxidate conditions. Both cases aimed at studying the effect on improvement of the effluent quality (mainly nitrogen and phosphorus removal). In Phase II, the lack of bottom saturation was maintained for VF-N. An acclimation period of one month was used in order to allow the development of biofilm as well as plant establishment.

Water samples were collected at the influent and effluents of the VF-N and VF-M. The physic-chemical parameters were monitored every two weeks by taking grab samples. pH, temperature (T), oxidation reduction potential (ORP), electrical conductivity (EC), COD, TSS, NH_4_^+^-N, NO_3_^−^-N, TN, PO_4_^−3^-P, total coliforms and *E. coli,* samples were taken, and transported, refrigerated, and analyzed at the water quality university Lab-UCM upon arrival. Transportation time was less than 30 min.

To evaluate water balance (water loss by evapotranspiration) and calculate evapotranspiration (ETP), influent and effluent water volume was measured for each loading period. Meteorological information (air temperature) was obtained from MeteoChile using the UC-Maule meteorological station [28].

To assess plant biomass development in the VSSF TWs, physiological characteristics of the plants such as height, number of leaves, and chlorophyll content of the leaves were measured every week using non-destructive methods (optical method). Due to the leaf thickness of *Schoenoplectus californicus*, chlorophyll content was measured at a point 10 cm before leaf ending. Once the experiment was completed, the biomass was assessed (leaves and roots) as well as N and P content in tissue (proximate analysis). One individual plant was taken in its entirety (leaves and roots) from one experimental unit of the three composing the VF-N and VF-M, while for the other two experimental units composing VF-N and VF-M, the plants were cut down to 0.50 m aboveground. This procedure follows the methods of Araya et al. [4].

### 2.4. Analytical Methods

The influent and effluent samples were filtered using a fiber glass filters with a 0.7 µm pore size. The physicochemical parameters COD, NH_4_^+^-N, NO_3_^−^-N, TN, and PO_4_^−3^-P were measured photometrically by a HI83399 multiparameter photometer (Hanna, Woonsocket, RI, USA) using reagent test kits as follows: COD, HI-93754B (medium range), NH_4_^+^-N, HI-93715 (medium range), NO_3_^−^-N, HI-93728 (medium range), TN, HI-93767 (low range), and PO_4_^−3^-P, HI-93713 (low range) and HI-93717 (high range) [21]. These determinations are modifications of standard procedures from APHA-AWWA-WEF [29]. TSS were analyzed gravimetrically according to the procedures in APHA-AWWA-WEF [29]. pH, T, ORP, and EC were measured with specific electrodes using a multiparameter portable Hanna HI 98194 instrument. The pathogens, total coliforms, and *E. coli* were analyzed using the Colilert simplified method [30]. Water volume was measured using a graduated cylinder.

Plant development was measured by tape measure; the number of leaves was manually counted [31]. Chlorophyll content was measured using a portable SPAD-502 Plus (Konica Minolta, Osaka, Japan) city, state abbrev if USA, country) [32,33].

The aboveground and belowground biomasses were measured after being dried in an oven at 80 °C for 24 h [4,14]. Proximate analysis considered the determination of P by calcination and colorimetry and the determination of N by combustion. These two procedures were doing according to the framework laid out by Sadzawka et al. [34]. Samples were sealed and sent to the Agricultural Research Institute of Chile (INIA) for proximate analysis.

### 2.5. Statistical Analysis

Statistical analysis was performed using INFOSTAT with a significant level of α = 0.05 [35] to determine the significant influences of bed depth, media change, and partial saturation. For response variables pH, ORP, EC, T, COD, TSS, TN, NH_4_^+^-N, NO_3_^−^-N, and PO_4_^−3^-P effluent concentrations from the VSSF TWs were used. Data calculated for ETP and water loss was also compared. The data was subject to Shapiro-Wilk normality test, to determinate the statistical test for comparison. Then, to determine the effects of bed depth, media change, and partial bottom saturation, VF-N and VF-M were compared for each Phase using a *t*-test for data with normal distribution or Wilcoxon test for data without normal distribution. The same statistical test was employed to determinate the effect of partial saturation in VF-M (Phase I vs. Phase II), that it’s means using a *t*-test for data with normal distribution or a Wilcoxon test for data without normal distribution.

## 3. Results and Discussion

### 3.1. Operational Conditions by Treatment System and Phase

HLR was applied uniformly during the experiment time: (a) VF-N, Phase I: 118.8 ± 5.5 mm/d, Phase II: 122.4 ± 2.7 mm/d, (b) VF-M, Phase I: 120.5 ± 5.9 mm/d, Phase II: 114.7 ± 2.6 mm/d. In other hand, Table 2 shows the operational conditions for VF-N and VF-M in each operational phase. The average pH for both treatment wetlands systems and phases ranges between 6.9 and 7.5, and therefore, classified as neutral, although significant differences (*p* < 0.05) can be found between the two phases for the two treatment wetlands systems. However, values between 6.0 and 8.0, similar to those found in this study, have been reported by other authors for VSSF TWs planted with ornamental and common plants species [14,36]. Thus, pH values are not expected to affect the removal process for organic matter (COD), nutrients (NH_4_^+^-N and PO_4_^−3^-P), and plant development [37].

Average temperature decrease was significant (*p* < 0.05) from Phase I to Phase II at, 5.6 °C for VF-N and 6.1 °C for VF-M. The temperature reduction can be explained by the time during which the experiment took place, which was between end of autumn and austral winter. This matches the climatic conditions of Mediterranean. The ORP average was over +100 mV for all the samples, the two TWs (with non-significant differences (*p* > 0.05) between them), and the two phases (VF-N and VF-M). These ORP values (>+100 mV) are in accordance with results from other VSSF TWs operated with sequential feeding schemes (fed by pulses) [3] and indicate that aerobic conditions were present in VF-N and VF-M [38]. The reduction of bed depth showed no significant effect (*p* > 0.05) (VF-M) on ORP values, and also, bottom saturation showed no significant effect (*p* > 0.05) (VF-M) on ORP conditions. Conversely, EC values indicate the presence of dissolved ions at a range similar to potable water (max. 1055 µs/cm) and tap water (500–800 µs/cm) [39]. In this case, the EC decreased 4.3% and 5.5% for VF-N and VF-M, respectively, between the two phases, but showed no significant (*p* > 0.05) difference.

### 3.2. Removal Efficiencies by Treatment System and Phase

#### 3.2.1. Organic Matter, Solids, Phosphorus, and Nitrogen Removal

Table 3 shows effluent concentrations and removal efficiencies of COD, TSS, NH_4_^+^-N, TN and PO_4_^−3^-P for VF–N and VF–M during the operational phases I and II. The COD effluent concentration increases and removal efficiencies decreased in significantly (*p* < 0.05) when going from Phase I to Phase II. However, average effluent concentrations were below 75 mg/L and removal efficiencies exceeded 60% for the two treatment systems (Table 3). This removal capacity is explained by the high oxidizing conditions present in the TWs that can cope with organic matter (ORP > +100 mV, Table 2). The fact that the removal was similar for the two mesocosms suggests that bed depth reduction and bottom saturation did not influence the VF-M treatment unit. TSS effluent concentration and removal efficiency were always below 10 mg/L and above 90%, respectively, for both treatment systems. The TSS removal capacity is explained by the physical filtration that was developed in the two VSSF TWs [40], which shows not effect of bed depth reduction and bottom saturation in the VF-M. The behavior of COD and TSS by the VF-N and VF-M are in agreement with that reported in the literature [1,41].

In terms of phosphate, VF-N showed significantly better results (*p* < 0.05) in comparison to VF-M for the two experimental phases with an average effluent concentration of above 0.5 mg/L and 30% higher removal efficiencies. This result shows that bed depth has an influence on phosphate removal, suggesting that precipitation and adsorption onto media play important role, despite that VF-N did not involve reactive media like zeolite [42]. High phosphorus removal rates can occur during the early periods when no reactive media is employed, as was the case in this study [43]. During Phase II, VF-N showed an increase of close to 1 mg/L in average effluent concentration while the VF-M showed an increase of only 0.5 mg/L. However, the increase was not significant in either case (*p* > 0.05). Despite that, the increase in effluent concentration for VF-N in comparison to Phase I shows that the material is to be saturated and is expected that P removal capacity is being reached. In the case of VF-M, the P saturation process of the non-reactive media at the top of VF-M would start in a way similar to VF-N, but the effluent concentration can potentially be maintained by the bottom saturation of the zeolite layer. However, the phosphate removal efficiency of 33% (for Phase II, with bottom saturation) was lower in comparison with other treatment wetlands that employ zeolites as support media, where removal efficiencies of up to 70% can be achieved [14]. One explanation for this difference could be the need for a longer residence time between wastewater and zeolite. For this experiment, the theoretical hydraulic retention time (HRT) was almost 0.5 days, taking zeolite’s porosity of 0.4 with HLR of 120 mm/d into account. This HRT is low in comparison to that of Vera et al. [14], who reported PO_4_^−3^-P removal efficiencies of around 70%, but with an HRT of 3.5 days. According to Vera-Puerto et al. [21], for Chilean natural zeolites, similar to those employed in this study, the time for achieving the 50% of the maximum adsorption capacity for PO_4_^−3^-P for granulometry of 1.0–3.0 mm and initial concentrations of 10 mg/L (similar conditions of this experiment), the HRT must be around 1.25 days (approximately 30 h).

**Figure 2:**
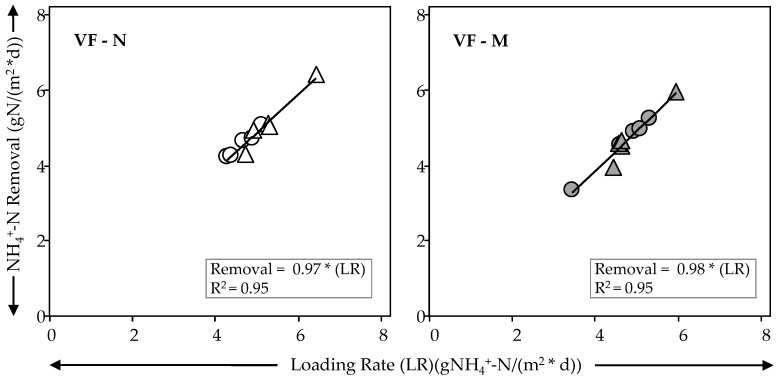
Relationship between NH_4_^+^-N loading rate and removal by treatment wetland and Phase. VF-Normal: Phase I (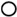), Phase II (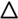); VF –Modified: Phase I (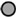), Phase II (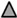).

Regarding NH_4_^+^-N in both treatment wetlands and phases, there were not significant differences (*p* > 0.05) in terms of effluent concentrations. The almost complete removal of ammonium (>95%) transformed to nitrate (Table 3) was achieved during the whole experiment time. This is, once again, the result of the prevalent aerobic conditions (ORP > +100 mV, Table 2) in both treatment wetlands as expected from VSSF TWs [44,45]. The removal of ammonium was achieved despite the reduction in bed depth of VF-M. Millot et al. [46], working with VSSF TWs, showed that around 60% of ammonium was removed in the first 0.20 m of depth while 75% of ammonium was removed at 0.40 m. Similar results were achieved by Arias et al. [47]. Thus, the results for VF-M achieved in this work and by Millot et al. [46], show the possibility of bed depth reduction for traditional VSSF TWs in terms of ammonium removal. In addition, Figure 2 shows that the almost 100% removal of ammonium is a linear function in terms of loading for both treatment wetlands (similar positive slope, VF-N and VF-M). Figure 2 also shows that, despite the inclusion of zeolite at the bottom in VF-M (only taking into account Phase I) and bottom saturation (Phase II), the removal of NH_4_^+^-N was consistent and no limit was reached during the test. The results for VF-M show that the transformation of ammonium into nitrate is developed during the first 0.25 m (Table 3), regardless of the modifications (media or saturation) at the bottom of the system.

The TN effluent concentration and removal efficiencies in Phase I showed similar behavior in that there was non-significant difference (*p* > 0.05) between VF-M and VF-N. However, during Phase II, when bottom saturation in VF-M was introduced, significant difference in TN effluent concentration (*p* < 0.05) between the two treatment wetlands can be seen. Additionally, effluent concentrations for VF-M had significant difference (*p* < 0.05) between Phase I and II. TN effluent concentrations to VF-M during Phase II were below 16 mg/L, the lowest value achieved in this study (Table 3). In addition, during Phase II for VF-M, effluent concentrations of NO_3_^−^-N were reduced significantly (*p* < 0.05) when compared with values from Phase I, and VF-N. Previous results show the positive influence of partial saturation on TN removal and are in agreement with the results of Silveira et al. [10], Oliveira et al. [48], and Pelissari et al. [49], who all employed partial saturation with the goal of improving TN removal. In addition, the TN removal efficiencies of around 60% achieved by VF-M during Phase II are similar to those achieved by Pelissari et al. [49], who showed removal efficiencies of 58% for VSSF TWs of a 0.70 m bed depth and partial saturation of 0.20 m. However, it can be hypothesized for this study that TN removal above 60% was not possible due to the lack of organic carbon need to complete the denitrification process. Furthermore, taking into account that the transformation of NH_4_^+^-N into NO_3_^−^-N was developed in the unsaturated layer of VF-M, the TN removal by VF-M during Phase II was increased by transformation of NO_3_^−^-N into N_2_ and not by ammonium adsorption properties of the zeolite layer. This is important because it shows that the zeolite layer can be replaced by traditional media like sand for TN removal. However, further research can be developed regarding increasing the height of the saturation layer and the replacement of zeolite in a similar way to the work of Silveira et al. [10], who modified in significant way (*p* < 0.05) effluent concentrations of NO_3_^−^-N when partial saturation was increase from 0.15 m to 0.25 m.

#### 3.2.2. Pathogens Removal

Table 4 shows removal of pathogens by each treatment wetland and Phase. The VSSF TWs provide some disinfection, and the objective of this section is showing the contribution regarding pathogen indicators performance of each of treatment lines [23,50]. VF-N had more than 50% of data as compared to VF-M when total coliforms and *E. coli* were analyzed at below 1 × 10^3^ (1000) MPN/100 mL for each phase. 1 × 10^3^ (1000) MPN/100 is a standard limit of discharge and/or reuse of effluents included in different guidelines and regulations [25,51].

VF-N showed an increase of 1.5 log units removal of total coliforms and 1.0 log units removal of *E. coli* as compared to VF-M for both experimental phases. In VSSF TWs removals between one and three log units of pathogens (fecal coliforms or *E. coli*)) have been reported [23,50]. These values are similar to results of the Table 4. Pathogens removal in VSSF TWs can be explained by the fact that the most frequent and well-validated removal mechanisms include natural die-off due to starvation or predation, sedimentation, filtration and adsorption; but filtration would be the main pathway [52]. Thus, the results in Phase I (no bottom saturation) showed that reduction in height of VF-M had effects on pathogen removal, confirming filtration as the main pathway. In Phase II saturation of the zeolite layer in VF-M did not have positive effect on the reduction of the effluent pathogen concentration because log removal units decreased as compared to Phase I. Additionally, there was no data below 1 × 10^3^ MPN/100 mL for this phase. Despite the fact that natural zeolite has a porous structure with valuable physicochemical properties, such as cation exchange (sodium, potassium, or calcium) molecular sieving, catalysis, and sorption [19,53], the results in Table 4 illustrate that properties of the natural zeolite have not an influence on pathogen removal when used as media in vertical treatment wetlands with or without partial saturation. This fact can be explained by the need for a longer residence time between wastewater and media as it was proposed by Torrens et al. [54]. Recently, Ezzat and Moustafa [55] reported positive influence of zeolite as media in horizontal treatment wetlands for pathogens removal with a theoretical HRT of 2.93 d, which is six times higher than theoretical HRT of this work (0.5 d).

Finally, despite that influent wastewater taking into account its concentration was in the range of “moderated” [8], previous results of the two experimental systems (VF-N and VF-N) for organic matter, solids, phosphorus, nitrogen, and pathogens removal suggest that is possible to treat even “concentrated” wastewater according to the range of concentration proposed by Henze et al. [8].

### 3.3. Effect of VSSF TWs Modifications in Plants Development

**Figure 3:**
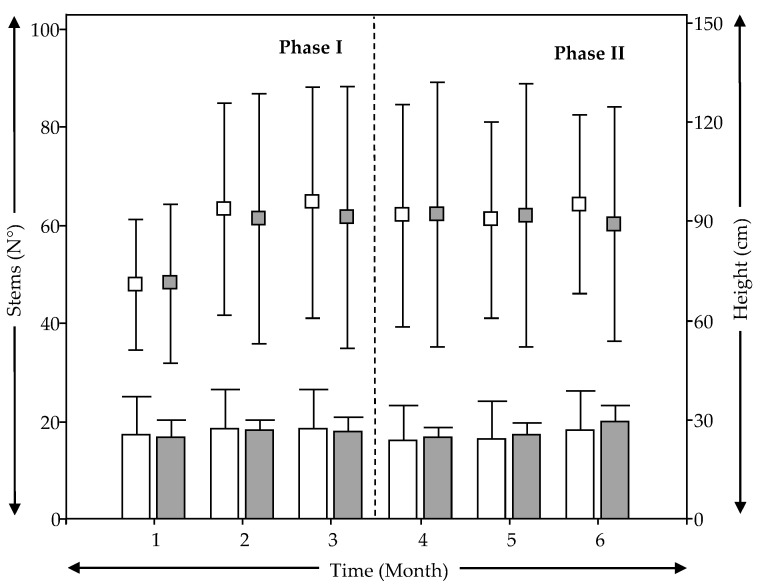
Behavior of plants during the experimental period for each treatment wetland and phase (bar chart: stems; dot chart: height). (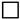) VF- N, (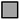) VF-M.

Figure 3 shows the development of the plants (*Schoenoplectus californicus*) for VF-N and VF-M over the course of the research time using the physiological characteristics of height (dot chart) and number of stems or leaves (bar chart). Figure 3 shows that *Schoenoplectus californicus* maintained stable growth in both treatment wetlands, with heights of around 0.90 m. This result is similar to that Rojas et al. [43], who achieved 0.90 m of height in six months of growing that included both the winter and spring seasons. Hidalgo-Cordero and García-Navarro [56] indicated that this plant can reach a height of up to 2.4–3.0 m in natural conditions. However, the height differences can be attributed to the fact that the experimental time covered the austral autumn and winter seasons, which are not a growing period for these plants. Another explanation is proposed by Alarcon et al. [57], who indicated that the growth and propagation of a macrophyte is favored if the wetland has enough free space and abundant nutrients. Limitation of space could have had an effect on plant´s growth in this study (Ø = 0.20 m, for each VSSF TWs). Figure 3 also shows that plants in VF-M and VF-N had similar variability in terms of the number of leaves, around 20. In this way, and taking into account physiological characteristics in Figure 3, plants developed similarly independent of bed depth reduction, media change, and bottom saturation in VSSF TWs during the first austral autumn and winter season.

**Figure 4:**
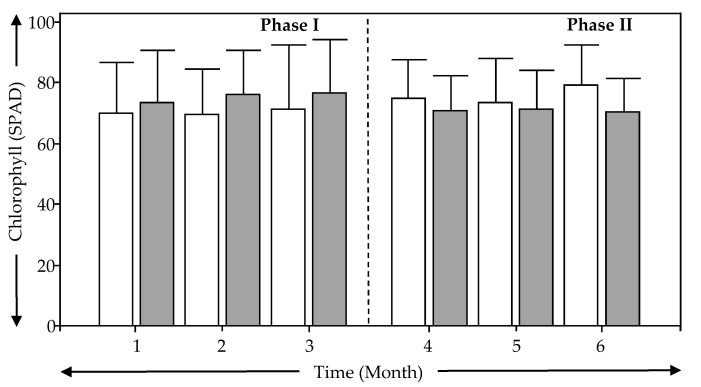
Evolution of chlorophyll status for each treatment wetland system and phase. (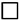) VF- N, (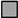) VF-M.

Figure 4 shows the evolution of the chlorophyll content measured in the leaves of the experimental units. VF-N and VF-M showed very similar average chlorophyll content during Phase I and Phase II with values around of 70 SPAD. This indicates regular activity in the plants despite the difference seasons. The chlorophyll content of leaves is often used to predict the physiological condition of the leaves, as influenced by various natural and anthropogenic factors, and indicate good both plant stress, and plant nitrogen (N) status [32]. Therefore, the results in Figure 4 show that modifications in bed depth, bottom saturation, and media change in VSSF TWs did not affect the plant *Schoenoplectus californicus* during the first austral autumn and winter of operation. This complements the results in Figure 3.

Table 5 shows the total biomass production by each treatment wetland during the experimental period. VF-N and VF-M showed similarity in terms of total biomass production (roots and leaves) with values of around 11 kgDW/m^2^-yr (DW: dry weight). This is similar to the results of Adhikari et al. [58], who report a biomass production between of 3.8 and 11 kgDW/m^2^-yr for *Schoenoplectus californicus* growing in natural and treatment wetlands. However, the results differ from those of Vera et al. [14], who reported values ranging from 0.64 to 1.70 kgDW/m^2^-yr. The difference can be explained by the fact that the biomass in Table 5, corresponds to addition of the three experimental units composing VF-N and VF-M. Conversely, the results of Vera et al. [14] correspond to only one experimental unit. In comparison to *Phragmites australis*, for TWs operated under similar climate conditions of this study, biomass production of 0.4 kgDW/m^2^-yr was reported; low in comparison to results of this study [59]. In addition, López et al. [59] after three years of operation concluded that the establishment of *Schoenoplectus californicus* was better, and is less vulnerable than *Phragmites australis* likely to be attacked by aphids. However, the two plant species contribute in similar way to nitrogen and phosphorus removal by TWs: below 10% of the total applied load. Considering the results presented in Table 5, *Schoenoplectus californicus* also contributed below 10% of the nitrogen and phosphorus removal for both experimental treatment systems: VF-N and VF-M.

Table 5 also shows that biomass of the leaves and roots differed between VF-N and VF-M at a rate below of 50%. This difference can be explained by a better development of the roots in VF-N due to more depth (1 m) as compared to VF-M (0.5 m). Despite that, leaf development was not affected, and was actually even better in VF-M. Results from Figure 3, Figure 4, and Table 5 shows that the growth and development of *Schoenoplectus californicus* was not affected by the modifications in VF-M during the first austral autumn and winter of operation, showing the possibility of this plant being used in modified VSSF TWs.

### 3.4. Behavior of Evapotranspiration and Water Loss in the Treatment Systems

**Figure 5:**
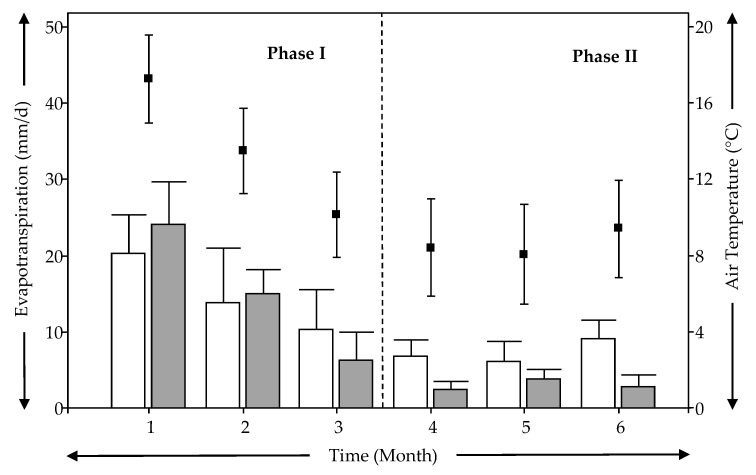
Evolution of evapotranspiration (ETP) (bar chart) during experimental time and relationship with mean monthly air temperature (dot char). (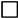) VF-N, (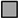) VF-M.

Figure 5 shows the average evapotranspiration and air temperature during the experimental time. Table 6 shows the evolution of average water loss during the experimental time. Measures of flow in the effluents showed a variability between 10% to 15% and reductions regarding to water applied in similar way that values of Table 6.

Figure 5 and Table 6 show that values of ETP and water loss follow the same pattern as average temperature on the monthly scale. In addition, average ETP and water loss (ETP, VF-N = (14.8 ± 6.9) mm/d, VF-M = (15.2 ± 8.2) mm/d, from Figure 5; water loss, Table 6) for the two experimental units (VF-N and VF-M), showed significant differences (*p* < 0.05) between Phase I and II. This was expected because ETP is the main process responsible for water loss in TWs and depending on the climate conditions as well as the plant growth stage [22,60]. Since the plant growth stage was the same for both VF-N and VF-M (Figure 3 and Figure 4), the effect of the meteorological conditions would be the prevalent factor responsible for water loss in the experimental VSSF TWs. Average ETP was always below 30 mm/d (Figure 5), and specially, the average values for winter were always below 10 mm/d. These values for winter fit with the findings of Pedescoll et al. [60], who reported similar results for different plant species used in treatment wetlands in a Mediterranean climate similar to the one in this study. Other authors, such as Filho et al. [61] and Headley et al. [22] reported ETP below 20 mm/d for horizontal subsurface flow treatment wetlands, similar to the values achieved in this study. For Phase I, VF-N and VF-M showed similar behavior in terms of ETP and water loss with non-significant differences (*p* > 0.05). Thus, the effect of bed depth reduction in VF-M was not significant (*p* > 0.05) regarding the water balance. However, in Phase II, VF-N and VF-M showed significant differences (*p* < 0.05). This result shows a significant effect (*p* < 0.05) on water loss for the system that was saturated at the bottom (VF-M), and this showed the lowest values for water loss and ETP as compared to those of VF-N. These results should be analyzed carefully because water behavior has to be tracked over longer periods, especially during the plant’s growing period of spring-summer, in order to obtain more conclusive results. This is because the bottom saturation of VF-M is expected to result in the water having more time into the treatment system. There is, therefore, possibility of increased the quantity of water lost by evapotranspiration when temperature and plant growth increases

## 4. Conclusions

Effluent water quality, especially in regards to organic matter, solids, and ammonium, suggests that it is possible to reduce the bed depth in VSSF TWs by 50%, if these pollutants are targeted to be removed. However, phosphate and pathogen removal would also be affected. Partial saturation had a positive influence only in terms of total nitrogen removal. Introduction of the natural zeolite into the saturated layer was not effective in terms of enhancing nutrient (nitrogen and phosphate) and pathogen removal.

*Schoenoplectus californicus* has the potential to be used in wetlands with modifications in bed depth, even if bottom saturation is used. Water balance showed that reduction in bed depth was not significant in terms of water loss. On the contrary, partial saturation had a positive effect by reducing water loss.

Finally, bed depth reduction and partial saturation, as modifications to VSSF TWs, planted with *Schoenoplectus californicus*, can be introduced to this technology, for rural wastewater treatment in austral Mediterranean conditions. However, zeolite layer in the bottom of VSSF TWs, accompanied by bed depth reduction and partial saturation, could be evaluated over a longer period of monitoring, to determinate its replacement by media commonly used in VSSF TWs, such as sand, that would help to reduce construction costs.

## Figures and Tables

**Table 1:** Wastewater characteristics. (*n* = 12).

Water Quality Parameter	Unit	Average ± Standard Deviation	Minimum	Maximum
T	°C	13.8 ± 4.5	8.8	24.5
pH		7.8 ± 0.2	7.3	8.1
EC	µs/cm	978 ± 87	866.0	1125.0
COD	mg/L	186.0 ± 62.0	125.0	325.0
TSS	mg/L	205.0 ± 228.3	30.5	658.3
NH_4_^+^-N	mg/L	43.0 ± 5.0	37.3	53.4
NO_3_^−^-N	mg/L	0.8 ± 0.5	0.2	1.6
TN	mg/L	50.0 ± 19.7	37.6	102.4
PO_4_^−^^3^-P	mg/L	9.3 ± 3.5	5.2	15.7
Total Coliforms	Log_10_(MPN/100 mL)	6.5 ± 0.6	5.0	7.0
*E. coli*	Log_10_(MPN/100 mL)	6.0 ± 0.5	5.0	6.6

**Table 2:** pH, temperature (T), electrical conductivity (EC) and oxidation reduction potential (ORP) by treatment wetland and phase. *n* = 6 for each phase.

Parameter	Unit	VF-N		VF-M	
		Phase I *	Phase II *	Phase I *	Phase II **
pH		7.4 ± 0.2	6.9 ± 0.3	7.5 ± 0.4	7.0 ± 0.1
T	°C	16.4 ± 4.5	10.8 ± 1.6	16.7 ± 4.3	10.6 ± 1.5
ORP	mV	+154.3 ± 69.9	+195.2 ± 71.4	+139.2 ± 73.5	+160.8 ± 107.0
EC	µs/cm	806.3 ± 105.2	772.2 ± 305.6	852.5 ± 94.2	805.3 ± 82.2

*: No bottom saturation; **: bottom saturation. T: temperature; ORP: oxidation-reduction potential; EC: electrical conductivity.

**Table 3:** Removal efficiencies by operational phase and treatment wetland. *n* = 6 for each phase.

Parameter	Effluent Concentrations (mg/L) and Removal Efficiencies (%)			
	VF-N		VF-M	
	Phase I *	Phase II *	Phase I *	Phase II **
COD	24.4 + 10.4 (83.9 ± 7.7)	68.5 ± 46.5 (65.1 ± 21.1)	45.8 ± 17.1 (73.6 ± 10.7)	74.5 ± 28.4 (60.9 ± 14.5)
TSS	1.7 ± 1.1 (97.1 ± 3.6)	2.4 ± 2.3 (98.2 ± 1.0)	5.4 ± 3.4 (94.1 ± 6.5)	6.5 ± 3.7 (93.5 ± 3.6)
PO_4_^−3^-P	2.2 ± 1.3 (80.6 ± 9.6)	3.3 ± 0.8 (51.4 ± 20.2)	4.1 ± 1.2 (61.8 ± 7.0)	4.6 ± 1.3 (33.3 ± 22.3)
NH_4_^+^-N	0.6 ± 0.4 (98.6 ± 0.9)	3.5 ± 4.3 (96.2 ± 2.8)	0.6 ± 0.4 (98.6 ± 0.9)	2.3 ± 2.7 (96.9 ± 3.8)
NO_3_^−^-N	37.2 ±13.3	46.8 ± 16.5	27.1 ± 8.8	19.5 ± 17.9
TN	43.5 ± 31.1 (23.9 ± 30.0)	36.8 ± 9.7 (19.1 ± 33.1)	37.1 ± 25.2 (35.3 ± 13.9)	15.5 ± 13.6 (63.3 ± 33.1)

*: not bottom saturation; **: bottom saturation. Number in parentheses shows removal efficiency. In the case of NO_3_^−^-N no removal efficiencies are reported due to increase of this compound in effluents.

**Table 4:** Total coliforms and E. coli effluent concentrations and removal by treatment wetland and operational phase. *n* = 6 for each phase.

Wetland Type	Phase	Total Coliforms			*E. coli*		
		Log. Units Removed	Data ≤ 1.0 × 10^3^ MPN/100 mL (%)	Min–Max	Log. Units Removed	Data ≤ 1.0 × 10^3^ MPN/100 mL (%)	Min–Max
VF-N	I *	3.3	90.0	2.0 × 10^2^–1.2 × 10^4^	3.0	100.0	1.0 × 10^3^
	II *	3.0	40.0	1.0 × 10^3^–2.2 × 10^5^	2.4	80.0	1.0 × 10^3^–2.3 × 10^5^
VF-M	I *	1.7	10.0	1.0 × 10^3^–3.7 × 10^5^	1.9	18.2	1.0 × 10^3^–1.8 × 10^5^
	II **	1.4	0.0	4.6 × 10^4^–>2.4 × 10^6^	1.1	0.0	1.2 × 10^4^–>2.4 × 10^6^

*: not bottom saturation; **: bottom saturation.

**Table 5:** Biomass and nutrient content by treatment wetland.

Wetland Type	Plant Tissue	Biomass (kgDW/m^2^ * Year)	Nutrient Content (%)	
			N	P
VF-N	Leaf	4.54 ± 0.05 *	1.69 ± 0.09	0.24 ± 0.03
	Root	7.06 ± 0.07 **	1.45 ± 0.07	0.22 ± 0.02
VF-M	Leaf	6.22 ± 0.07 *	1.62 ± 0.08	0.24 ± 0.02
	Root	5.20 ± 0.05 **	1.63 ± 0.08	0.25 ± 0.03

* Three individuals. ** One individual.

**Table 6:** Percentage of water loss by treatment wetland and phase.

Phase	Month	Water Loss (%)	
		VF-N	VF-M
I	1	13.6 ± 3.0 *	16.2 ± 3.2 *
	2	9.8 ± 5.3 *	10.5 ± 2.4 *
	3	7.3 ± 3.8 *	4.4 ± 2.6 *
	Average	10.2 ± 4.7	10.4 ± 5.4
II	4	5.1 ± 1.8 *	1.9 ± 1.0 **
	5	4.4 ± 1.9 *	2.7 ± 0.9 **
	6	6.5 ± 1.7 *	2.1 ± 1.1 **
	Average	5.3 ± 1.9	2.3 ± 1.0 **

*: not bottom saturation; **: bottom saturation.
